# Short-term follow-up of intestinal flora in radiation-exposed mice

**DOI:** 10.1093/jrr/rrz002

**Published:** 2019-04-11

**Authors:** Kanako Yamanouchi, Takakiyo Tsujiguchi, Yamato Sakamoto, Koichi Ito

**Affiliations:** 1 Department of Bioscience and Laboratory Medicine, Graduate School of Health Sciences, Hirosaki University, 66-1 Hon-cho, Hirosaki, Japan; 2 Department of Radiation Science, Graduate School of Health Sciences, Hirosaki University, 66-1 Hon-cho, Hirosaki, Japan

**Keywords:** intestinal flora, *Bifidobacterium*, *Lactobacillus*, alimentary tract disorder

## Abstract

Some gastrointestinal bacteria, otherwise known as the ‘intestinal flora’, can cause severe gastrointestinal problems, including sepsis, which are strongly linked to lifestyle-related diseases, including cardiovascular diseases. Several investigations have focused on the long-term changes in the intestinal flora associated with radiation exposure; however, the short-term effects remain unknown. In this study, we tracked the short-term changes in the intestinal flora of mice exposed to different doses of X-ray irradiation (2 Gy and 4 Gy), focusing only on the lactic acid bacteria *Bifidobacterium* and *Lactobacillus*. A decrease in the *Lactobacillus* abundance was detected immediately after irradiation in individuals exposed to both 2 Gy and 4 Gy irradiation. However, mice exposed to 4 Gy of irradiation showed a remarkable increase in *Bifidobacterium*, indicating a potential role of these bacteria in regeneration of the intestinal epithelial tissue. Studies on changes in intestinal bacteria as a result of radiation exposure are limited. Therefore, continuation of this field of research is expected to provide important fundamental insight into the mechanisms by which radiation causes damage to the intestinal tissues, contributing to the development of sepsis.

## INTRODUCTION

The human mucosa occupies a substantial portion of the body surface area, including the oral cavity, nasal cavity, gastrointestinal tract, respiratory organs, and genital organs, accounting for an area more than 200 times that of the skin surface. Among the components of the mucosa, the digestive tract contributes a particularly big area, largely due to the presence of microvilli on the surface of the intestinal folds, villi, and epithelial cells, so as to absorb as many nutrients as possible through expanding the area [1]. In addition, various bacteria coexist on the mucous membranes of mammals. Indeed, recent reports indicate that as many as 100 trillion bacteria exist in the human intestinal tract, representing a cell population that is several times greater than that of the human host somatic and germ cells [2, 3]. These symbiotic bacteria of the gut are collectively referred to as the ‘intestinal flora’, and accumulating evidence points to a close association between the composition of the intestinal flora and host health, including the onset of disease [4, 5].

Exposure to high-dose radiation results in remarkable disturbances to tissues and organs with high regenerative ability, such as the hematopoietic tissue and intestinal mucosa [6]. However, the intestinal tract, rich in epithelial stem cells, shows high radiosensitivity, and the consequent collapse of the intestinal barrier leads to dissolution of body fluids containing electrolytes, along with infiltration of intestinal bacteria into the blood circulation [7]. Such transmigration of intestinal bacteria to the blood is referred to as bacteremia, which can progress into severe sepsis, leading to systemic symptoms such as multiple organ failure and ultimately death. For example, women exposed to 5.7 Gy radiation in the Goiania radiological accident in Brazil were reported to have suffered from septicemia [8]. In addition, the intestinal bacterial flora has been found to become dominated by *Bacteroides* spp. in patients receiving pelvic irradiation treatment, regardless of the presence or absence of diarrhea [9]. These findings indicate that radiation destroys the balance of the intestinal bacterial flora. Thus, one strategy to improve the symptoms induced by such intestinal injury is to restore the balance of the intestinal flora and maintain the barrier function of the intestinal mucosa. However, the specific changes in the intestinal bacteria and intestinal mucosa that occur in an individual immediately after radiation exposure remain unclear.

In this study, we exposed mice to X-ray irradiation, and compared the abundance of the genera *Bifidobacterium* and *Lactobacillus* before and after irradiation in order to evaluate the short-term effects on the composition of the beneficial bacteria comprising the intestinal flora. These genera were chosen because they are lactic acid bacteria that decompose sugars into lactic acid so as to prepare the intestinal environment.

## MATERIALS AND METHODS

### Animal experimentation

Seven-week-old C57BL/6Njcl female mice (CLEA Japan, Inc., Japan) were used as the experimental model. After an acclimatization period of 1 week after acquisition, the 8-week-old mice were irradiated with 2 Gy and 4 Gy X-rays using an MBR-1520 R-3 X-ray irradiation apparatus (Hitachi, Japan). Irradiation conditions were 1 Gy/min, with a tube voltage of 150 kV, tube current of 20 mA, and a Al 0.5 mm + Cu 0.3 mm filter. Three mice were used in each experimental group. To monitor the health condition of the mice throughout the experiment, body weight and food intake were measured every day for 2 weeks after irradiation. Feces were collected before irradiation, and at 1, 2, 6, 12, 24, 48 and 72 h after irradiation for analysis of intestinal flora with real-time polymerase chain reaction (PCR). The animals were maintained in a temperature-controlled room at 23.9 ± 0.3°C with 26.6 ± 4.2% humidity. Moreover, the mice were periodically inspected for the presence of pathogens (bacteria, viruses, parasites) to confirm maintenance of a specific-pathogen-free environment. The animal experiments were conducted in compliance with guidelines concerning the use of laboratory animals of Hirosaki University (approval number: G 17003).

### Real-time PCR of intestinal bacterial flora

NucleoSpin® DNA Stool (TAKARA BIO INC., Japan) was used to extract the DNA of feces. Three PCR primers sets were prepared to analyze the changes in the abundance of intestinal bacterial flora (Table 1) [10–12]. Analysis was performed by detection of excitation light with a Power SYBR® Green Master Mix (Thermo Fisher Scientific Inc., Waltham, MA, USA) on the Step One Plus instrument (Applied Biosystems Inc., Foster City, CA, USA). Each cycle threshold (CT) value of the target bacteria was corrected by the CT value for the ‘all bacteria’ PCR product, and compared with the respective CT value before irradiation to determine the relative abundance. All target CT values were corrected by the ΔCT method and compared with the 0 hour value (ΔΔCT method). The PCR conditions were 40 cycles of thermal denaturation at 95°C for 15 s, annealing for 30 s, and elongation at 80°C for 30 s. The annealing temperature was set for each primer as indicated in Table 1. CT values of all fecal DNA samples were measured three times in triplicate.

**Table 1.** Table showing the targeted bacterial taxa, bacterial genes and their primers used for qPCR assays

Phyla/Division	Family	Primer (5′–3′)	Production length (bp)	Annealing temp (°C)
**All bacteria**		GCCTAACACATGCAAGTCGA	472	58
		GTATTACCGCGGCTGCTGG		
**Actinobacteria**	**Genus*****Bifidobacterium***	AGGGTTCGATTCTGGCTCAG	156	58
		CATCCGGCATTACCACCC		
**Firmicutes**	**Genus*****Lactobacillus***	TGGAAACAGRTGCTAATACCG	232	62
		GTCCATTGTGGAAGATTCCC		

### Statistical analysis

For statistical analysis of the bacterial flora, the Student’s *t*-test was performed as a relative comparison between the values before and after irradiation with Origin Ver. 8.1 statistical analysis software.

## RESULTS

### Weight fluctuations and food intake of experimental mice

**Fig. 1.**
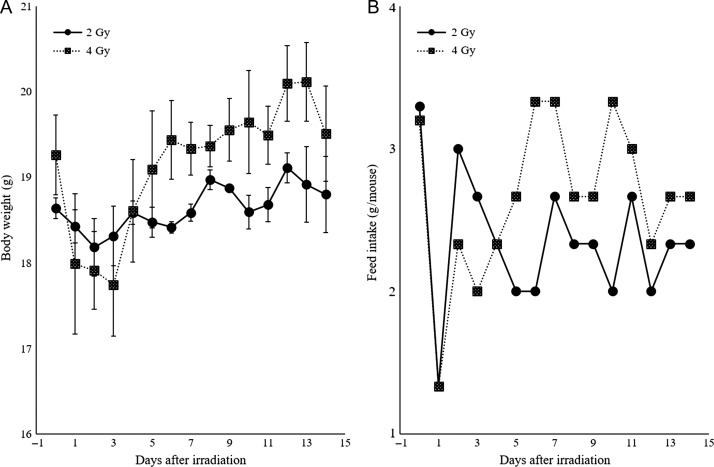
Body weight fluctuation and feed intake of irradiated mice. (A) Body weight decreased from 1 to 3 days after irradiation in both the 2 Gy– and 4 Gy–irradiated groups. (B) A sharp decline in food intake was observed after 1 day of irradiation followed by recovery.

Food intake decreased sharply at 24 h after irradiation, and temporary weight loss was observed on the first day after irradiation. Thereafter, both the 2 Gy– and 4 Gy–irradiated groups showed an increase in food intake and body weight, and no individuals died during the 2-week observation period (Fig. 1).

### Short-term fluctuations of the intestinal bacterial flora upon irradiation exposure

**Fig. 2.**
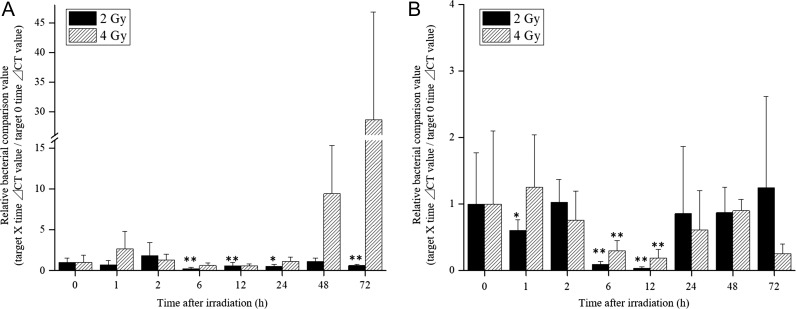
Changes in intestinal flora in radiation-exposed mice. The asterisks indicate a significant difference (*P < 0.05, **P < 0.01) when compared with the control group (0 h). (A) *Bifidobacterium* significantly decreased in the 2 Gy–irradiated group 6 h after irradiation, but showed a sharp increase from 48 h after irradiation in the 4 Gy–irradiated group, which continued until 72 h. (B) Both groups showed a sharp decrease in *Lactobacillus* from 6 to 12 h after irradiation and then recovered.

Quantification of naturally discharged fecal bacteria showed changes in both *Bifidobacterium* and *Lactobacillus* (Fig. 2). In the 2 Gy–irradiated group, *Bifidobacterium* showed a decreasing trend from 6 h after irradiation, which continued until 72 h. However, the abundance of *Bifidobacterium* increased by 9-fold in the 4 Gy–irradiated group as of 48 h after irradiation, and reached a 28-fold increase compared with the pre-irradiation level after 72 h (Fig. 2A). Moreover, the expression of *Lactobacillus* genes decreased from 6 h to 12 h after irradiation, and then recovered up to the baseline level in both groups (Fig. 2B). This finding suggests that 2 Gy or 4 Gy irradiation might result in short-term suppression of intestinal bacterial growth, but the normal bacterial flora will be rebalanced. Interestingly, the sharp increase in *Bifidobacterium* with the higher dose (4 Gy) of irradiation was confirmed. Although there are individual differences, *Bifidobacterium* in the 4 Gy–irradiated group increased ~10 times after 48 h and reached 28 times after 72 h.

## DISCUSSION

The two bacterial genera targeted in this study, *Lactobacillus* and *Bifidobacterium*, are known to exhibit various probiotic effects, and have thus been adopted in the treatment of gastrointestinal diseases in clinical practice as probiotics [13, 14]. For example, *Lactobacillus rhamnosus* GG strain (LGG) is used for the treatment of traveler’s diarrhea, antibiotic-associated diarrhea, and relapsing *Clostridium difficile* colitis [15]. *Bifidobacterium bifidum* has also been reported to have a beneficial effect on the clinical course of rotavirus diarrhea [16]. These effects are attributed to several mechanisms of the bacteria, such as resistance to acids and bile, adhesion to intestinal cells, and regulation of the mucosal immune response, thereby contributing to improvement of diarrhea and pathogen reduction [15, 17]. Thus, in recent years, it has become clear that the probiotic activity of *Lactobacillus* and *Bifidobacterium* plays a major role in stabilization of the barrier function of the intestinal mucosa.

The normal intestinal mucosa functions as a barrier (intestinal barrier) that eliminates bacteria attempting to invade intestinal tissue and cells. However, in an inflamed, pathogenic, or radiation-induced intestinal tract disorders, the osmotic pressure of the barrier changes, allowing intestinal bacteria to invade the blood and other organs [18, 19]. Oral administration of a probiotic to living bodies subjected to such gastrointestinal disturbances has been shown to influence the intestinal permeability and contribute to normalization of the intestinal microflora, with eventual stabilization of the intestinal environment [20]. In addition, oral administration of *Lactobacillus* to irradiated mice is shown to prolong survival and suppress the incidence of sepsis [21].

Although few studies have examined the variation of intestinal bacteria in radiation-exposed individuals, there is some evidence that radiation leads to an increase in bacteria known to be hazardous to the host [22–24]. Intestinal damage due to radiation in humans is recognized as the most frequent side effect of cancer treatment to the pelvic organs [25–27]. A study on the changes in the intestinal bacterial flora in patients who received pelvic irradiation treatment shows that the radiation therapy led to deterioration in the intestinal environment due to an increase in *Bacteroides* spp., regardless of the presence or absence of diarrhea [9]. In addition, exposure to radiation above 10 Gy led to intestinal death in rats, which was accompanied by an increase in Proteobacteria comprising gram-negative pathogenic species [28]. Proteobacteria is a phylum that contains many pathogenic bacteria such as *Salmonella*, *Vibrio*, and *Helicobacter*, and thus an increase in the abundance of this phylum increases the risk of sepsis [29–31].

Cellular exposure to ionizing radiation leads to oxidizing events that alter atomic structure through direct interactions of radiation with target macromolecules or via products of water radiolysis [32–34]. In general, radioresistant bacteria are thought to have enzymes, such as catalase and superoxide dismutase, to neutralize active oxygen species produced *in vivo*, and to aid DNA regeneration and repair [35–37]. In this study, *Lactobacillus* without catalase and superoxide dismutase seems to have caused a transient decrease. *Bifidobacterium*, which showed an increase in this study, can produce catalase and superoxide dismutase. Therefore, *Bifidobacterium* may be radioresistant and may show a relative increase so as to supplement other intestinal bacteria transiently suppressed proliferation. In addition, several studies have reported that some *Bifidobacterium* strengthen the barrier function of the intestinal tract and prevent migration of pathogenic bacteria and foreign substances into the blood [38–40]. Therefore, increased *Bifidobacterium* in radiation-exposed mice may be effective in restoring radiation-impaired intestinal epithelium. Furthermore, *Bifidobacterium* produces lactic acid and acetic acid by glycolysis and shows the action of decreasing intestinal pH and regulating the intestinal environment, so *Bifidobacterium* promotes the recovery of a radiation-injured intestinal epithelium injured and the associated disordered intestinal environment. It may recover the intestinal flora, including *Lactobacillus*, which had decreased. However, this study is a relative comparison study, and therefore this result does not reflect an actual increase or decrease in bacterial groups, so continuation of this research is necessary.

## FUNDING

This work was supported by the Japan Society for the Promotion of Science (JSPS) KAKENHI [Grant Number JP 18K15578].

## CONFLICT OF INTEREST

The authors have no conflict of interest to declare.
